# Antitumor effects of ^32^P-chromic-poly (L-lactide) brachytherapy in nude mice with human prostate cancer

**DOI:** 10.3892/ol.2013.1443

**Published:** 2013-07-03

**Authors:** LIUJING SUN, XISHAN ZHU, LONGBAO XU, ZIZHENG WANG, GUOQIANG SHAO, JUN ZHAO

**Affiliations:** 1 Department of Urinary Surgery, Changzhou No. 3 People’s Hospital, Jiangsu 213000, P.R. China; 2 Department of Nuclear Medicine, Changzhou No. 2 People’s Hospital, Changzhou, Jiangsu 213000, P.R. China; 3 Department of Clinical Nuclear Medicine, Nanjing No. 1 Hospital, Nanjing, Jiangsu 210006, P.R. China

**Keywords:** ^32^P-chromic-poly (L-lactide), prostate cancer, brachytherapy

## Abstract

The aim of the present study was to investigate the antitumor effects and tissue distribution of ^32^P-chromic-poly (L-lactide) (^32^P-CP-PLLA) in nude mice with human prostate cancer. Tumor models were obtained by transplantation of PC-3M tumor cells into male BALB/c nude mice. Animals were randomly divided into control, ^32^P-chromic phosphate (^32^P-CP) colloid and ^32^P-CP-PLLA groups (all n=20). A series of indices were investigated, including apoptosis of tumor cells, rate of apoptosis, expression of caspase 3 and 8, biodistribution and intratumoral concentration of ^32^P-CP-PLLA, intensity of radioactivity, tumor volume and microvessel density (MVD). Highly concentrated radioactivity of ^32^P-CP-PLLA in the tumor mass was detected by single photon emission computed tomography (SPECT) scanning. The residual activities of the ^32^P-CP-PLLA and ^32^P-CP colloid groups were 3.02±0.32 and 1.76±0.31 MBq, respectively, on day 14 following treatment. The tumor inhibition rates were 67.24±3.55 and 55.92±7.65%, respectively (P<0.01). Necrotic changes, in conjunction with apoptosis, were observed in the treatment group. MVD values for the ^32^P-CP-PLLA and ^32^P-CP colloid groups were 28.24±10.07 and 36.15±11.06, respectively. ^32^P-CP-PLLA showed an excellent capacity for killing tumor cells, inducing apoptosis and inhibiting angiogenesis.

## Introduction

Prostate cancer is ranked second among cancer-related mortalities in American male citizens (1). Radiotherapy, with or without endocrine therapy, remains the preferred treatment for the majority of patients with localized prostate cancer. The principle of radiotherapy is to improve the dose of radioexposure in tumor tissues. Generally, the external irradiation dose for conventional radiotherapy is 65–70 Gy, while those of three-dimensional conformal and intensity modulated radiation therapy are ~80 Gy (2–4). Brachytherapy, a term used to describe radiation treatment, exhibits a higher irradiation dose since the radiation source is put in direct contact with the tumors. Previous studies have communicated an equivalent treatment efficiency, as well as reduced trauma and side effects, following brachytherapy when compared with that of radical surgery and external beam radiotherapy in patients with prostate cancer (5,6).

At present, low dose rate brachytherapy, for example, permanent low dose irradiation via transplantation of seeds, including ^125^I, is the preferred treatment for low risk prostate cancer in a number of countries and the outcome for moderate- and high-risk prostate cancer patients remains satisfactory (7–9). However, adverse effects, including bone marrow depression and migration via the blood circulation, are frequently reported due to transplanted seeds remaining *in vivo* permanently (10,11).

^32^P has been recognized as the ideal therapeutic radionuclide for its unique characteristics, including a pure β-particle emitter with a physical half-life of 14.3 days and a maximum and average energy of 1.71 and 0.695 MeV, respectively. A number of radioactive drugs, pharmaceuticals in the form of colloid and microspheres, are hypothesized to represent promising drugs for the treatment of solid tumors. ^32^P-chromic phosphate (^32^P-CP) colloid has been applied for the treatment of intracavitary cancer (12) and its efficiency has been shown to be satisfactory following interstitial injection (13). However, a number of studies have indicated that toxicity of the liver, spleen and bone marrow may be induced due to the transmigration of the colloid (14). In addition, ^32^P-CP colloid has been identified as a comparatively safe and convenient procedure for the treatment of refractory solid tumors (15–17), however, solutions to the following obstacles remain to be identified: i) enhancement of the local biological effects of ^32^P by increasing the dosage; ii) control of the distribution of microspheres or colloid outside the tumor mass; and iii) reduction or even elimination of toxicity and side effects. In addition, the complexities associated with dose calculation and clinical practice prevent the development of ^32^P-CP colloid for clinical use. Thus, the identification of a novel vector for the transportation of ^32^P radionuclide is crucial for low dose rate brachytherapy.

Poly (L-lactic acid) (PLLA) has been widely used as a drug delivery system due to its excellent biocompatibility and biodegradability (18–20). It is a thermoplastic aliphatic polyester derived from renewable resources and is capable of biodegradation under specific conditions. In the present study, ^32^P-CP-PLLA microparticles were produced with these characteristics and degraded continuously under specific temperatures and humidities. In addition, a comparative study was performed in nude mice with human prostate cancer to investigate the differences in the pharmacokinetic profile and treatment efficiency in ^32^P-CP colloid and ^32^P-CP-PLLA groups.

## Materials and methods

### Cell culture

PC-3M human prostate cancer cells (Nanjing KeyGen Biotech Co., Ltd., Nanjing, China) were maintained in stationary monolayer cultures at 37ºC, with 5% CO_2_ in a humidified atmosphere, using Roswell Park Memorial Institute medium supplemented with 10% heat-inactivated fetal bovine serum (Nanjing KeyGen Biotech Co., Ltd.) and L-glutamine. A total of 90 healthy male BALB/c nude mice (Shanghai Laboratory Animal Research Center, Shanghai, China) at 4–6 weeks old and 18–22 g were maintained in Streamline^®^ cabinets (Streamline Laboratory Products, Changi, Singapore) at 25–27ºC and a humidity of 40–50%.

### Drug administration

^32^P-CP-PLLA was prepared as described previously (21). Briefly, 100 mg PLLA (0.1 μm in diameter) was added to 1 ml sterile ^32^P-CP colloid (radiochemical purity, >98%; Beijing Atom High Tech, Beijing, China) and dehydrated alcohol was used as a dispersant. The affinity between PLLA and the colloid was modulated by surface-active agents. The mixture was treated by ultrasonication for 30 min, kept at room temperature for 1 h and dried in the drying vacuum oven at 60ºC. Pentobarbital (2%; 0.1 ml) was injected via peritoneal injection following anesthesia. The paracentesis needle was inserted into the center of the tumor along the long axis, followed by injection with microparticles. The radioactivity concentration was 0.39 GBq/ml (10.5 mCi/ml) and the needle was removed once resistance was felt. Following dilution with a physiological solution of sodium chloride, the intratumoral injection activity was 7.4 MBq (0.05 ml) for the colloid.

### Preparation of animal models

A subcutaneous inoculation of 2×10^6^ PC-3M cells on the right upper flank was performed to induce tumorigenesis. Continuous measurements of tumor dimensions were conducted using a caliper. Tumor volume was calculated by the following formula: Tumor volume = length/2 × width^2^.

Animals were randomly divided into ^32^P-CP colloid (7.4 MBq, intratumoral injection), ^32^P-CP-PLLA (7.4 MBq, intratumoral injection) and control (equivalent volume of saline) groups when tumor volume reached 8 mm in diameter. All animal experiments were carried out according to national laws. This study was approved by the ethics committee of Changzhou No. 3 People’s Hospital.

### Single photon emission computed tomography (SPECT) imaging

Bremsstrahlung scintigraphy of ^32^P-CP-PLLA and ^32^P-CP colloid distribution was investigated using SPECT fitted with a low-energy, general-purpose collimator (Siemens, Erlangen, Germany). The single pinhole SPECT system was operated in a routine manner. In brief, a cylinder with a diameter of 25 mm, designed to be tight fitting for mice, was positioned directly and horizontally above the pinhole aperture. A mechanical support allowed for the precise and manual adjustment of the cylinder in two directions; the distance of the cylinder to the pinhole aperture, which equals the radius of rotation, and along the axis of the cylinder to select the field of view. The pinhole collimator was connected to an ADAC ARC 3000 scintillation camera (Philips Co., Ltd., Shanghai, China) and had a focal length of 320 mm and an opening angle of 60º. The energy window was set at 78 keV, 30% width and 30 min harvest time. SPECT images were captured at 1 and 12 h and 1, 2, 4 and 8 days post-administration.

### Treatment efficiency

Tumor volume was determined every two days following treatment. The tumor inhibition rate was calculated on day 14 using the following formula: tumor inhibition rate = (W1 – W2)/W1 × 100; where W1 and W2 represent the average weight of tumor volume in the control and treatment groups, respectively. For the calculation of the tumor inhibition rate, 5 mice were sacrificed in each group for conventional histological examination by formalin fixation and paraffin embedding. Hematoxylin and eosin staining was performed for the monitoring of sections.

### TUNEL assay

Apoptosis was measured using a TUNEL assay kit (Nanjing KeyGen Biotech Co., Ltd.) at 1, 6, 12, 24 and 72 h (each group, n=3) following treatment. The procedures were performed according to the manufacturer’s instructions. The apoptosis index was calculated as the ratio of apoptotic cells to total cells.

### Expression of caspase 3 and 8

Expression of caspase 3 and 8 was evaluated using caspase 3 and caspase 8 Activity Assay kits according to the manufacturer’s instructions (Nanjing KeyGen Biotech Co., Ltd.). The Bio-Rad Microplate 550 Reader (Bio-Rad, Hercules, CA, USA) was used to monitor the absorbance at 405 nm. The activities of caspase 3 and 8 were evaluated by the ratio of the optical density of the inducer to the negative control.

### Microvessel density

Slides stained with anti-CD34 monoclonal antibody were examined via microscope. The cells that were positive for CD34 were designated as a vessel and initially observed at ×100 magnification to select the high density areas of microvessels. Next, the microvessel density (MVD) of the CD34-positive cells was investigated at ×200 magnification.

### Radioactivity determination

A β-radioactivity meter was used to determine radioactivity and final radioactivity was calculated as described previously (22).

### Statistical analysis

All data are presented as mean ± standard deviation. Statistical analyses were performed using SPSS 13.0 Software (SPSS, Inc., Chicago, IL, USA) and statistical significance was detected by ANOVA. P<0.05 was considered to indicate a statistically significant difference.

## Results

Anorexia was identified in the control group and skin ulcers were detected in the right lower extremities of 2 mice. However, no anorexia or skin ulcers were observed in the treatment groups and tumor growth was reduced significantly compared with the control group. In the ^32^P-CP colloid group, irregular and eccentric growth of the tumor mass and local recurrence was identified in 1 mouse. No eccentric growth or recurrence was observed in the ^32^P-CP-PLLA group.

**Figure 1:**
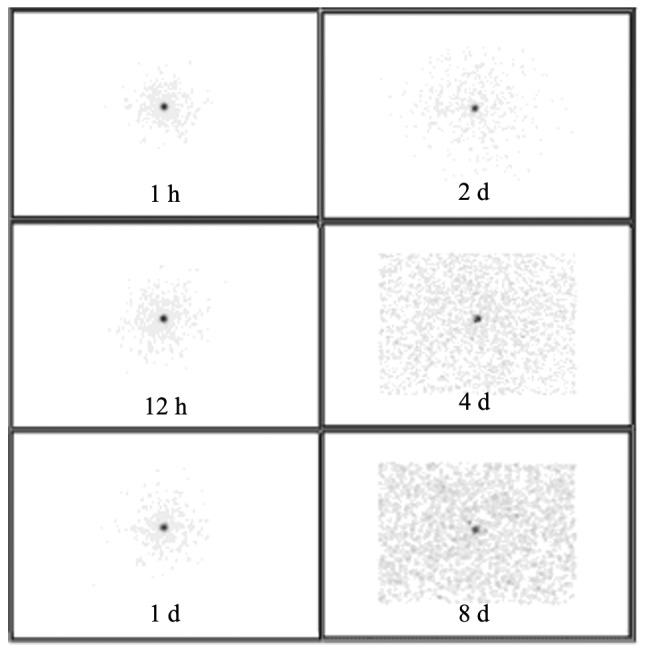
SPECT imaging of the ^32^P-CP-PLLA group at 1 and 12 h and 1, 2, 4 and 8 days post-administration, demonstrating a gradual reduction. SPECT, single photon emission computed tomography.

**Figure 2:**
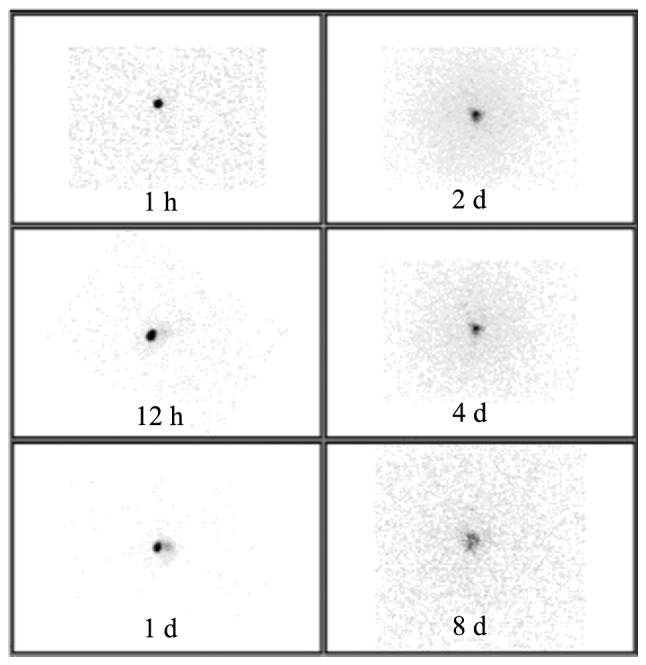
SPECT imaging of ^32^P-CP colloid group at 1 and 12h and 1, 2, 4 and 8 days post-administration, indicating that the colloid concentrated area was significantly reduced. SPECT, single photon emission computed tomography.

Following implantation of ^32^P-CP-PLLA microparticles, biodistribution was detected by SPECT imaging. During the initial stages, microparticles were concentrated at the implantation sites, however, over time, a low radioactive uptake (RAU) of ^32^P-CP-PLLA was identified in the tumor mass compared with the baseline levels. In addition, no shifting or loss of ^32^P-CP-PLLA was identified (Fig. 1). In the ^32^P-CP colloid group, a sharp decrease of ^32^P-CP colloid was identified following injection due to the absorption of the colloid by the peripheral tissues. Thus, a significant increase of RAU was identified in the peripheral tissues compared with the baseline levels (Fig. 2).

**Figure 3:**
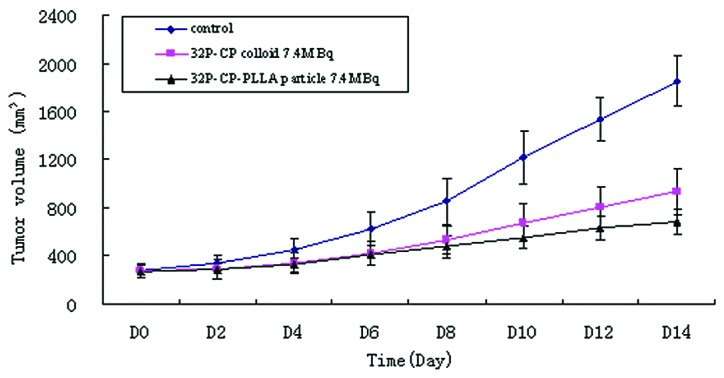
Tumor volume in control and treatment groups. ^32^P-CP-PLLA, ^32^P-chromic-poly (L-lactide).

**Figure 4:**
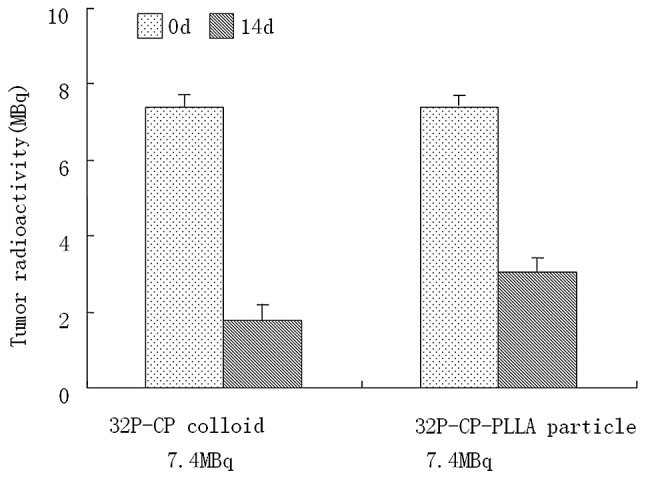
Comparison of radioactivity in microparticle and colloid groups. ^32^P-CP-PLLA, ^32^P-chromic-poly (L-lactide).

Delayed growth of the tumor mass was observed in the ^32^P-CP-PLLA group compared with the ^32^P-CP-colloid and control groups (Fig. 3). Significant differences were identified between the ^32^P-CP-PLLA and ^32^P-CP colloid groups and the tumor volume, the tumor inhibition rate and the ratio of necrocytosis on day 14 (P<0.01; Table I, Fig. 3). The residual activity on day 14 in the ^32^P-CP-PLLA and ^32^P-CP colloid groups was 3.02±0.32 and 1.76±0.31 MBq, respectively (Fig. 4).

**Figure 5:**
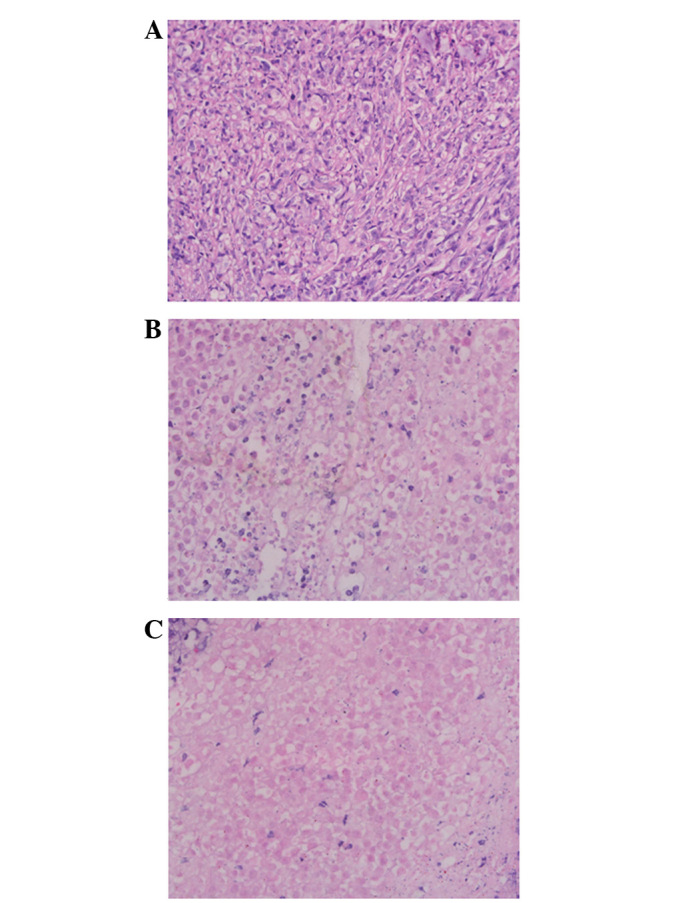
Pathological imaging of tumor masses in (A) control, (B) ^32^P-CP colloid and (C) ^32^P-CP-PLLA groups. ^32^P-CP-PLLA, ^32^P-chromic-poly (L-lactide). Hematoxylin and eosin staining; magnification, ×200.

With regard to pathological detection, various extents of necrosis were identified in the treatment groups. Tumor tissues in the control group exhibited karyomegaly and a high karyoplasmic ratio. Inflammatory cell infiltration was identified surrounding the ^32^P-CP-PLLA during the early stages and coagulation necrosis was observed at later stages. A significant amount of necrosis was observed in the ^32^P-CP-PLLA group compared with the ^32^P-CP-colloid group (Fig. 5).

**Figure 6:**
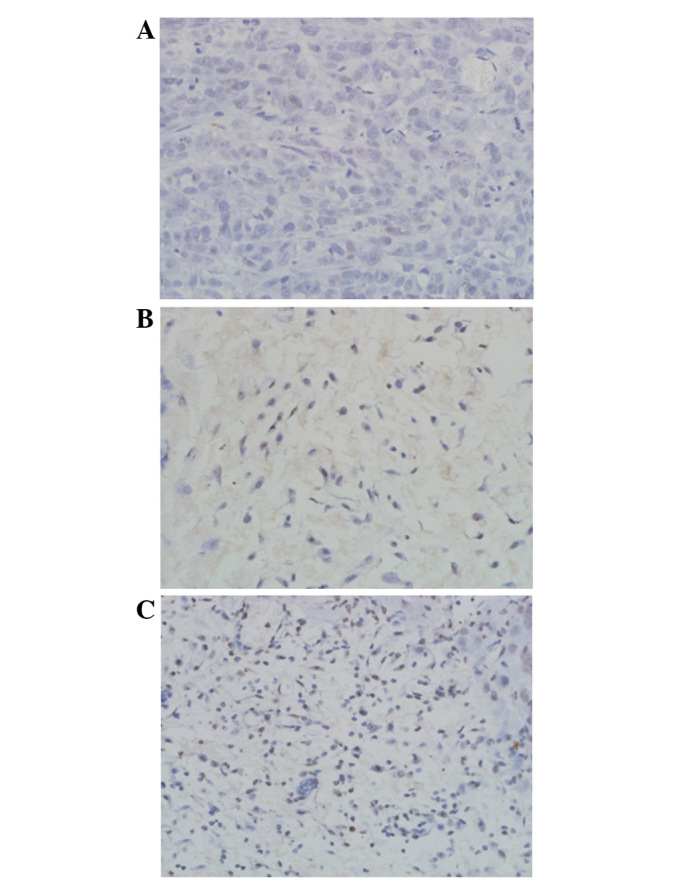
TUNEL assay in (A) control, (B) ^32^P-CP colloid and (C) ^32^P-CP-PLLA groups (DAB; magnification, ×200). ^32^P-CP-PLLA, ^32^P-chromic-poly (L-lactide). Hematoxylin and eosin staining; magnification, ×200.

**Figure 7:**
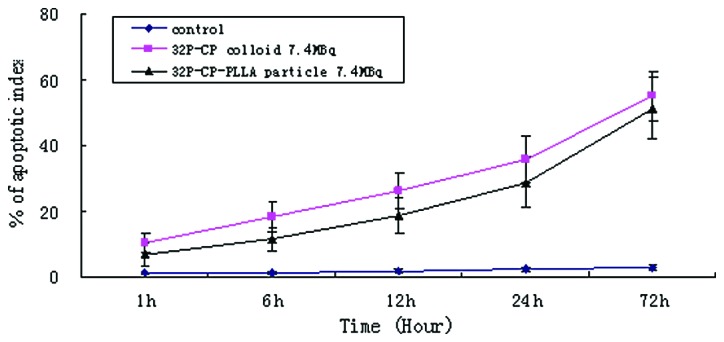
Plot of the apoptotic index in control and treatment groups. ^32^P-CP-PLLA, ^32^P-chromic-poly (L-lactide).

The TUNEL assay indicated a large amount of irregular apoptosis with nuclear debris and apoptotic bodies in the treatment groups (Fig. 6). The rate of apoptosis increased progressively with time compared with the control group (P<0.01; Fig. 7). In addition, significant differences in caspase expression were identified in the treatment groups at 6, 12 and 24 h following administration compared with the control group (P<0.01; Table II), however, no significant differences were identified at 1 and 72 h.

**Figure 8:**
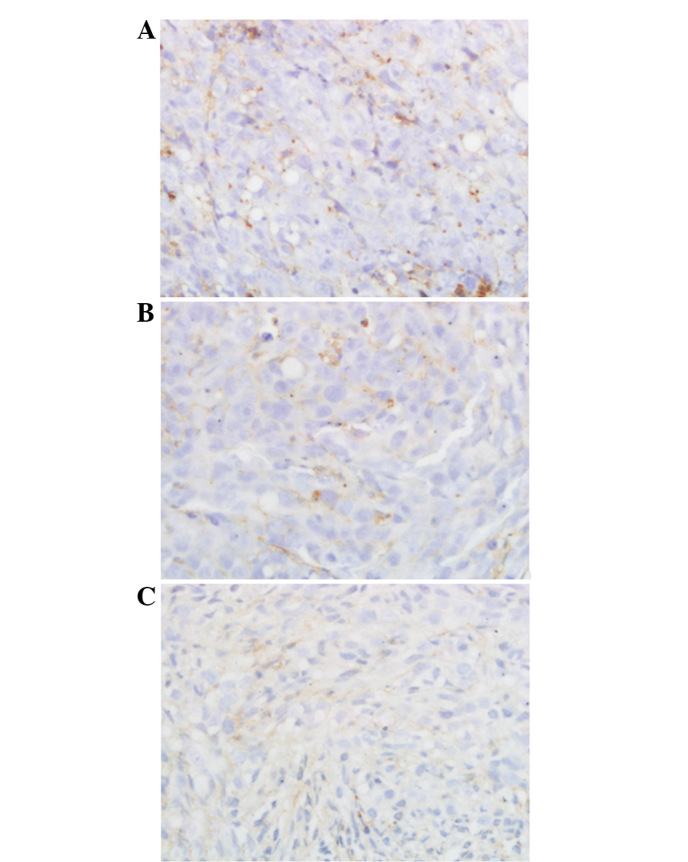
MVD in (A) control, (B) ^32^P-CP colloid and (C) ^32^P-CP-PLLA groups. ^32^P-CP-PLLA, ^32^P-chromic-poly (L-lactide). Hematoxylin and eosin staining; magnification, ×200.

A significant difference in MVD was identified among the treatment and control groups (P<0.01) with MVD values of 60.71±8.21, 36.15±11.06 and 28.24±10.07 for the control, colloid and microparticle groups, respectively (Fig. 8).

## Discussion

Previously, ^32^P has been hypothesized to represent the ideal radionuclide for brachytherapy. In the present study, PLLA was used as the delivery vector for ^32^P-CP to modulate the target orientation and safety of internal radiation therapy. PLLA has excellent biodegradation and biocompatibility and was approved as a pharmaceutical adjuvant by the FDA in 1997. PLLA is now widely used as a drug delivery system (18).

In the present study, once the ^32^P-CP colloid was administered by interstitial injection, it was immediately absorbed by the tumor mass via the capillary vessels, lymphatic system and tissue space and diffused into the peripheral tissues. However, the distribution of ^32^P-CP colloid in the tumor mass was not uniform due to certain differences, including the morphous of the injection channel in the tumor mass, colloid leakage from the injection orifice, tissue density and the abundance of capillaries. When compared with ^32^P-CP colloid, ^32^P-CP-PLLA with identical radioactivity carried an equal amount of ^32^P-CP-colloid and was able to reduce the amount of ^32^P-CP colloid passing through the lymphatic system, capillaries and tissue space by gradual disintegration. This resulted in improved drug retention and uniform distribution of ^32^P-CP-PLLA in the tumor mass. SPECT imaging identified that the microparticles were limited to the tumor mass due to the low RAU of the peripheral tissue. A marked decrease of ^32^P-CP-colloid was identified as the colloid was absorbed by the peripheral tissues and the remaining radioactive intensity of ^32^P-CP-PLLA was higher compared with that of the ^32^P-CP colloid on day 14. Previously, Yang *et al* reported that the biodistribution of ^32^P-CP-PLLA and ^32^P-CP colloid was 9.3 MBq in Balb/c nude mice implanted with BxPC-3 human pancreatic tumors, with concentrations of ^32^P-CP-PLLA and ^32^P-CP colloid at 241.73±131.06 and 170.61±69.01% ID/g, respectively (21). These observations indicated that ^32^P-CP-PLLA shows higher retention effects and is capable of enhancing the therapeutic effects while decreasing the blind zone of radiotherapy in addition to ^32^P-CP absorption.

It has been previously identified that cell proliferation and apoptosis are markedly associated with prostate cancer. In the present study, necrosis and apoptosis were identified in the tumor mass of the treatment groups, indicating that ^32^P-CP has the ability to kill tumor cells and induce apoptosis by emitting β-rays. A similar radiation effect (apoptosis rate) was identified between the ^32^P-CP colloid and ^32^P-CP-PLLA groups, therefore, indicating that ^32^P-CP-PLLA is likely to maintain its concentration in the tumor mass using the PLLA delivery system.

A previous study reported that angiogenesis was associated with lymph node metastasis, recurrence, remote metastasis and mortality rates due to prostate cancer (23). Currently, CD34 antibody immunohistochemistry is frequently applied as an indicator of MVD. The present results showed that MVD in the treatment groups was lower than that of the control group and that MVD values obtained from the peripheral tissue were higher than those from the central tumor mass. This demonstrated that the embolism and necrosis of blood vessels were induced by β-rays emitted by ^32^P. Lower MVD values were identified in the ^32^P-CP-PLLA group compared with the colloid group indicating that the microparticles may inhibit angiogenesis by improving the radioactive dosage in the tumor mass. Gellman *et al* reported that intimal proliferation is likely to be induced by a lower dose of internal irradiation (24). However, no intimal proliferation was identified in the present treatment group, indicating that ^32^P-CP-PLLA may inhibit the local recurrence of prostate cancer.

Two major pathways that have been identified as associated with apoptosis are the extrinsic and intrinsic pathways (death receptor and mitochondrial pathways, respectively). The extrinsic pathway is activated by ligand-activated death receptors, including Fas ligand (FasL). Caspase 8 functions as a significant initiation factor for apoptosis, with caspase 3 as the concluding indicator for apoptosis and the core molecule of the Fas/FasL signaling pathway (24). The results of the current study identified that the expression of caspase 8 and 3 showed a gradual increase and reached peak values at 6 and 12 h following treatment in the colloid and microparticle groups, respectively. Caspase activity subsequently showed a marked decrease due to cell necrosis and local radiation effects. Therefore, this demonstrated that caspase 3 and 8 are involved in apoptosis induced by β-radiation. In addition, peak levels of caspase 3 and 8 were obtained later in the ^32^P-CP-colloid group compared with the ^32^P-CP-PLLA group (6 vs. 12 h). This is likely to be associated with the temporarily high local dose in the ^32^P-CP colloid group during the early stages (~6–12h) compared with the gradual degradation of ^32^P-CP-PLLA.

The present results showed that ^32^P-CP colloid and ^32^P-CP-PLLA may kill tumor cells, induce apoptosis and inhibit angiogenesis. However, the tumor volume was reduced in the ^32^P-CP-PLLA group compared with the ^32^P-CP colloid group at 8 days post-treatment, and the most significant difference was identified on day 14. This demonstrated an enhanced retention time of ^32^P-CP-PLLA at the target site, and an accumulated yield of radiation was identified in the ^32^P-CP-PLLA group following the gradual delivery of the radioactive source of equivalent radioactivity levels. Therefore, this indicated that ^32^P-CP-PLLA functions via low dose and gradual delivery of the radioactive source.

Overall, the results demonstrated that ^32^P-CP-PLLA had the following distinct characteristics: i) enhanced metabolism of the drug at the target site and reduced dose distribution outside the tumor mass by degradation and delayed release of the drugs; ii) permanent stagnation and complications were avoided due to an excellent degradation capacity of the transplanted microparticles, under specific conditions; iii) biological metabolism of ^32^P-CP-PLLA was regulated by ^32^P-CP-PLLA activity *in vivo* with specific dosages convenient for individual therapy; and iv) as a polymer, modification of the structure of ^32^P-CP-PLLA was applicable and may aid the possible development of a delivery system with increased efficiency, including the synergic sensitization of chemotherapeutics.

With regard to the application of ^32^P-CP-PLLA microspheres in the clinical treatment of prostate cancer, the microspheres may be precisely implanted into prostate cancer lesions under the three-dimensional radiotherapy planning system. In addition, as the microspheres are likely to be degraded gradually over a specific period, repetitive implantation is possible to prevent the lymph node metastases and micrometastasis of prostate cancer. Therefore, ^32^P-CP-PLLA may decrease the recurrence of prostate cancer and improve the survival rates of patients. In addition, the range of β-array radiation emitted by ^32^P was ~4 μm and thus it may cause slight or no damage to sexual function and decrease the occurrence of urinary incontinence, urethral stenosis and rectal complications. It is therefore hypothesized that ^32^P-CP-PLLA may represent an innovative method for the treatment of prostate cancer.

## Figures and Tables

**Table I:** Comparison of treatment efficiency in the control and treatment groups on day 14.

Group	Radioactivity, MBq	Tumor mass, g	Tumor-inhibition rate, %	Necrosis in tumor mass,%
Control	0.0	1.62±0.21	-	4.92±4.25
^32^P-CP colloid	7.4	0.70±0.12^a^	55.92±7.65	62.58±7.59^a^
^32^P-CP-PLLA	7.4	0.53±0.06^a^,^b^	67.24±3.55^b^	75.82±3.24^a^,^b^

a P<0.01, vs. control;

b P<0.01, vs. ^32^P-CP colloid.

32 P-CP-PLLA, ^32^P-chromic-poly (L-lactide).

**Table II:** Expression of caspase 3 and 8 at specific time intervals.

	Caspase 3					Caspase 8				
		
Group	1 h	6 h	12 h	24 h	72 h	1 h	6 h	12 h	24 h	72 h
Control	0.96±0.05	0.98±0.04	1.02±0.06	1.06±0.05	1.04±0.06	1.00±0.04	1.02±0.04	1.06±0.02	1.04±0.05	1.06±0.06
^32^P-CP colloid	1.05±0.12	2.56±0.19	2.05±0.15	1.32±0.11	0.91±0.09	1.08±0.09	2.27±0.21	1.45±0.18	1.08±0.07	1.03±0.07
^32^P-CP-PLLA	1.06±0.08	1.53±0.13	2.21±0.14	1.37±0.11	0.93±0.12	1.06±0.13	1.58±0.21	2.12±0.22	1.07±0.14	1.02±0.05

32 P-CP-PLLA, ^32^P-chromic-poly (L-lactide).
